# A Randomized Controlled Trial of Thai Medicinal Plant-4 Cream versus Diclofenac Gel in the Management of Symptomatic Osteoarthritis of the Knee

**DOI:** 10.1155/2022/8657000

**Published:** 2022-06-13

**Authors:** Nut Koonrungsesomboon, Achariya Churyen, Supanimit Teekachunhatean, Chaichan Sangdee, Nutthiya Hanprasertpong

**Affiliations:** ^1^Department of Pharmacology, Faculty of Medicine, Chiang Mai University, Chiang Mai 50200, Thailand; ^2^Clinical Research Center for Food and Herbal Product Trials and Development (CR-FAH), Faculty of Medicine, Chiang Mai University, Chiang Mai 50200, Thailand; ^3^Musculoskeletal Science and Translational Research Center, Chiang Mai University, Chiang Mai 50200, Thailand; ^4^McCormick Faculty of Nursing, Payap University, Chiang Mai 50000, Thailand; ^5^Center of Thai Traditional and Complementary Medicine, Faculty of Medicine, Chiang Mai University, Chiang Mai 50200, Thailand

## Abstract

**Background:**

Osteoarthritis of the knee is a common degenerative musculoskeletal condition. Thai Medicinal Plant-4 (TMP-4) cream is made up of *Garcinia mangostana* peel, *Sesamum indicum* seeds, *Glycine max* (L.) Merr. seeds, and *Centella asiatica* leaves, all of which have anti-inflammatory and analgesic properties. The present study aimed at determining the efficacy and safety of TMP-4 cream versus diclofenac gel in the treatment of symptomatic osteoarthritis of the knee.

**Methods:**

A randomized-controlled trial was conducted to assess knee pain on a scale of 100 mm Visual Analog Scale (VAS) and other key metrics, including VAS knee stiffness, a modified 10-step stair climb test, a timed up and go test, the Knee Injury and Osteoarthritis Outcome Score, and safety outcomes, following administration of either TMP-4 cream or diclofenac gel for 4 weeks.

**Results:**

A total of 199 patients with moderate knee pain intensity were randomly assigned to either TMP-4 cream or diclofenac gel (allocation ratio 1 : 1). The mean changes of VAS knee pain in the TMP-4 cream and diclofenac gel groups were −31.68 ± 14.18 mm and −31.09 ± 12.41 mm, respectively, (mean difference = −0.58, 95% confidence interval = −4.37–3.20, *P*=0.761). The upper limit of 95% confidence interval for the comparison between TMP-4 cream and diclofenac gel was within the predefined margin of 7 mm for noninferiority. The safety was comparable between the two interventions.

**Conclusions:**

TMP-4 cream was noninferior to diclofenac gel in relieving osteoarthritic knee pain and may be considered as an alternative therapeutic option in the treatment of symptomatic osteoarthritis of the knee.

## 1. Introduction

Osteoarthritis of the knee is one of the most common degenerative musculoskeletal disorders, with nearly 650 million people suffering from this condition worldwide [1]. It represents a major public health problem worldwide, with an increasing trend in disease prevalence and burden in proportion to the growing number of elderly people [2]. Osteoarthritis of the knee is characterized by pain and diminished joint mobility and function, which place a significant economic burden on individual patients, health care providers, and society [3]. Nearly half of adults acquire symptomatic osteoarthritis of the knee by the age of 85 years, necessitating pharmacological treatment for appropriate pain control [4].

Nonsteroidal anti-inflammatory drugs (NSAIDs) remain the cornerstone of currently recommended pharmacologic therapy for the management of symptomatic osteoarthritis of the knee [5]. Several clinical practice guidelines agree on the benefits of topical NSAID therapy for patients with localized symptoms of knee osteoarthritis [6–9]. With repeated administration, topical NSAIDs can penetrate the osteoarthritic knee, accumulate in the tissues of the knee joint, and help to minimize inflammation [10, 11]. Evidence suggests that topical NSAIDs are effective in knee pain relief [12–14] and provide the same degree of pain alleviation and function improvement as oral NSAIDs in individuals with mild-to-moderate osteoarthritis of the knee [15–17]. Besides, topical NSAIDs have a better safety profile than oral NSAIDs due to their lower systemic exposure, with far fewer reports of systemic adverse drug reactions [18, 19]. Nowadays, topical NSAIDs are increasingly often preferred over oral NSAIDs for local pain management, especially in the elderly [20–22], and are now considered as first-line pharmacologic therapy for symptomatic treatment of osteoarthritis of the knee [6–9].

In response to the growing health and economic burden of osteoarthritis of the knee, alternative pain-relieving and mobility-improving therapies for osteoarthritic knees are presently receiving a lot of attention. In the current era, complementary and alternative medicine is commonly used for the management of chronic diseases, including osteoarthritis of the knee [23–27], and herbal extract-based formulations are likely to be a viable option for establishing such medicinal remedies in this case [28–30]. The Thai Medicinal Plant-4 (TMP-4) cream is made up of four plants: *Garcinia mangostana* peel, *Sesamum indicum* seeds, *Glycine max* (L.) Merr. seeds, and *Centella asiatica* leaves and is developed by the Thai Mangosteen Research & Development Center. All of the plant ingredients in TMP-4 cream have been found to have strong antiinflammatory and analgesic properties [31–37], so TMP-4 cream is supposed to offer pain-relieving qualities when given to patients with osteoarthritis of the knee. However, scientific evidence is required to provide a therapeutic claim.

The present study aimed at determining the efficacy and safety of TMP-4 cream and diclofenac gel in the management of symptomatic osteoarthritis of the knee by means of a randomized controlled trial.

## 2. Methods

### 2.1. Study Design and Setting

This prospective, randomized, single-blind, active-controlled, parallel-group, noninferiority trial was conducted at the Faculty of Medicine, Chiang Mai University, Chiang Mai, Thailand, between November 2017 and November 2018. The trial followed the OARSI Clinical Trials recommendations for the design, conduct, and reporting of clinical trials for osteoarthritis of the knee [38, 39], as well as the CONSORT 2010 guidelines for reporting parallel group randomized trials [40] and two other relevant extensions to the CONSORT statement, i.e., the recommendations for reporting randomized controlled trials of herbal interventions [41] and the recommendations for reporting of noninferiority randomized trials [42]. The clinical trial protocol and supporting documentation were approved by the Research Ethics Committee of the Faculty of Medicine, Chiang Mai University (No. 190/2017). This study was prospectively registered with the Thai Clinical Trials Registry (TCTR20171123002) prior to enrollment.

### 2.2. Study Participants

Patients with symptomatic osteoarthritis of the knee, as defined by the American College of Rheumatology [43], with Kellgren and Lawrence radiographic criteria of Grade 2 or higher, were eligible for this study. Inclusion criteria were as follows: age greater than 45 years, osteoarthritic knee pain for at least 3 months prior to enrollment, and of moderate pain intensity (as defined by a pain score of 35–75 mm on a 100 mm Visual Analog Scale (VAS) [44]), and ability to walk and climb stairs. All patients signed a written informed consent form.

Those who had any of the following criteria were excluded: (1) other underlying inflammatory arthropathies (e.g., rheumatoid arthritis and gouty arthritis), (2) signs or symptoms of acute flares in knee osteoarthritis (i.e., marked swelling, redness, warmth, and tenderness around the knee joint), (3) clinically significant knee joint effusion, (4) a schedule of knee surgery to be taken place in the next few months, (5) a recent knee injury, (6) skin disease around the afflicted knee, (7) a malignant tumor, (8) a history of hypersensitivity to NSAIDs or any ingredients in TMP-4 cream or diclofenac gel, or (9) clinically significant abnormalities in any of the following laboratory findings, i.e., hemoglobin <9 g/dL, white blood cells <4,000 cells/mm^3^, platelets <100,000 cells/mm^3^, alanine transaminase or aspartate transaminase >2 times the upper limits of normal, estimated glomerular filtration rate <45 mL/min/1.73 m^2^, and uric acid >9 mg/dL). The trial also excluded individuals who had received an intraarticular corticosteroid injection within the previous three months or who had used symptomatic slow-acting drugs for osteoarthritis (SYSDOA) (e.g., glucosamine sulfate, chondroitin sulfate, diacerein, and hyaluronan) for less than four months or who had stopped using these drugs within the previous six months, as well as those who were pregnant or breastfeeding.

### 2.3. Study Intervention and Comparator

TMP-4 cream was manufactured by Asian Phytoceuticals Public Company Limited (APCO). It consisted of four herbs: *G. mangostana* peel, *S. indicum* seeds, *G. max* seeds, and *C. asiatica* leaves. The components of TMP-4 cream are summarized in Table S1. Diclofenac gel was chosen to be a comparator in this trial because it had previously been shown in a network meta-analysis to be superior to a placebo for pain relief over four weeks of therapy [19]. Diclofenac gel (Voltaren® Emulgel®, 1% diclofenac gel, Reg. No. 1A 752/41) was purchased from OLIC (Thailand) Limited.

### 2.4. Randomization, Blinding, and Allocation Concealment

Computer-generated random numbers were obtained before trial initiation, with a 1 : 1 allocation using a block size of 10. Research personnel who had no clinical involvement in this study dispensed either TMP-4 cream or diclofenac gel according to the computer-generated randomization list. The allocation sequence was concealed from the investigator in sequentially numbered, opaque, sealed envelopes. Only after each participant had satisfied the eligibility requirements and completed all of the baseline tests at Week 0 were the corresponding envelopes unsealed. TMP-4 cream and diclofenac gel were in identical opaque plastic tubes.

Outcome assessors were kept blinded to the intervention group assignment of each patient. Even though the two interventions did not appear to be similar, the patients were not told of the formulation features of the intervention during the informed consent process. In this case, incomplete disclosure was intended to minimize the patient's performance bias, and such information was debriefed at the end of the study.

### 2.5. Study Procedures

**Figure 1:**
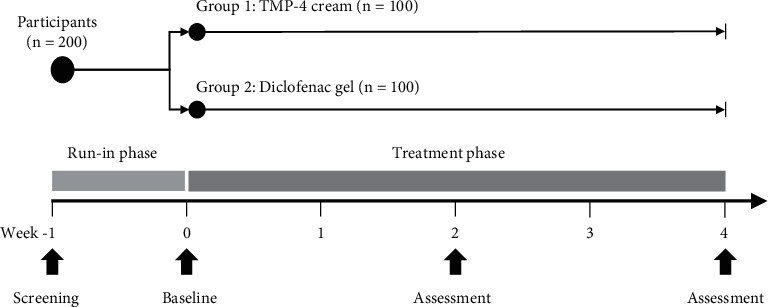
Study design. A four-week randomized active-controlled trial enrolled 200 patients with osteoarthritis of the knee, 100 of whom were randomly assigned to Group 1 (TMP-4 cream), and the other to Group 2 (diclofenac gel). Outcome assessments were carried out at baseline and the end of Week 2 and Week 4 of the treatment phase.

In this study, there was a one-week run-in phase followed by a four-week treatment phase (Figure 1). Following the screening, all patients who met the eligibility criteria were required to stop using any arthralgia/arthritis treatment modalities, including NSAIDs and other analgesics, as well as any other topical drugs/products in the area surrounding the afflicted knee, throughout the study period. The literature suggests that a one-week washout period is necessary to establish a true baseline [45].

At the beginning of the treatment phase (Week 0), eligible patients with moderate knee pain were randomly assigned to receive either TMP-4 cream or diclofenac gel, which was administered four times daily for four weeks. This trial protocol required the patients to avoid using any concurrent or rescue pain medication during study participation. Some patients might be prematurely withdrawn from the experiment if they had any of the following symptoms/conditions: (1) severe osteoarthritic knee pain requiring other medications or treatment modalities, (2) acute inflammation of the afflicted knee, (3) a knee injury, (4) use of other NSAIDs or analgesic drugs, (5) moderate-to-severe allergic or adverse drug reactions (or mild but not improving after appropriate treatment), and (6) lost to follow-up.

### 2.6. Outcome Assessment

Outcome assessment was performed at baseline (at the end of the one-week run-in phase) and at the end of Week 2 and Week 4 of the treatment phase (Figure 1). Efficacy outcome measures included

1. A horizontal 100 mm VAS knee pain score on a scale of 0 to 100, with a higher value indicating more severe knee pain [46]
2. A horizontal 100 mm VAS knee stiffness score on a scale of 0 to 100, with a higher value indicating more severe knee stiffness;
3. A modified 10-step stair climb test (mSCT), which involved ascending a flight of 10 stairs in a certain amount of time [47]
4. A timed up and go test (TUG), which consisted of standing up from a chair, walking 3 meters, turning, and returning to a sitting position [48]
5. The Knee Injury and Osteoarthritis Outcome Score (KOOS), which consisted of five dimensions (i.e., pain, other symptoms, activities of daily living, function in sports and recreation, and knee-related quality of life), each of which was rated on a 5-point Likert scale and transformed to a scale of 0 to 100, with a higher value indicating fewer knee problems [49, 50]

The patient's perception of overall improvement and the physician's assessment of overall improvement were also assessed using a horizontal 100 mm VAS, with a higher value indicating better improvement. Following nondirective inquiries, adverse events seen by the outcome assessor or self-reported by the patients were recorded. At each follow-up visit, the weight of the cream/gel that remained in the tubes was measured to determine medication compliance. Patients who used the intervention at less than 60% of the prescribed dosage were considered poorly compliant.

### 2.7. Study Endpoints

The primary efficacy endpoint was the change from baseline in knee pain as measured by a horizontal 100 mm VAS during the four weeks of therapy. In patients with bilateral knee osteoarthritis, the knee with more prominent symptoms at baseline was used as an index knee for the efficacy assessment. Responders were defined as individuals whose VAS knee pain decreased by at least 50% from their baseline value and by at least 20 mm in absolute terms [51]. This cut-off value is commonly used to represent the clinical importance of pain relief from the patient's perspective. Secondary endpoints included other efficacy outcome metrics and safety outcome measures.

### 2.8. Sample Size Determination

A total of 200 patients (100 in each group) were expected to be enrolled in this study. The sample size of 100 per group was estimated using a noninferiority margin of 7 [52, 53], assuming a mean difference (MD) of 0 and a standard deviation (SD) of 18 [54], with a precision and confidence level of 95%, 80% power, and a dropout rate of 15% [55].

### 2.9. Statistical Analysis

The per-protocol (PP) and modified intention-to-treat (MITT) approaches were used to assess the efficacy outcomes in this study. The PP analysis included only patients who completed the treatment regimen with adequate compliance during the course of four-week treatment. In the MITT analysis, the data of patients who had prematurely left the trial were estimated using the last observation carried forward technique. All patients who had received at least one dosage of the assigned intervention were assessed for the safety evaluation.

The Student's *t*-test and the Fisher's exact test were used for comparing mean differences and the distribution of dichotomous variables, respectively, between the two groups. A repeated-measures ANOVA with the least significant difference (LSD) test was applied to evaluate if there were any variations in the mean values of each variable between the baseline and the two successive follow-up visits. A comparison of the TMP-4 and diclofenac groups on VAS knee pain was undertaken to determine noninferiority, using a noninferiority margin of 7 mm [52, 53]. If the upper limit of the two-sided 95% confidence interval (95% CI) for the MD of VAS knee pain did not surpass a margin of 7 mm, noninferiority was declared. Statistical analysis was performed using SPSS version 22.0. Statistical significance was defined as a *p* value of less than 0.05.

## 3. Results

**Figure 2:**
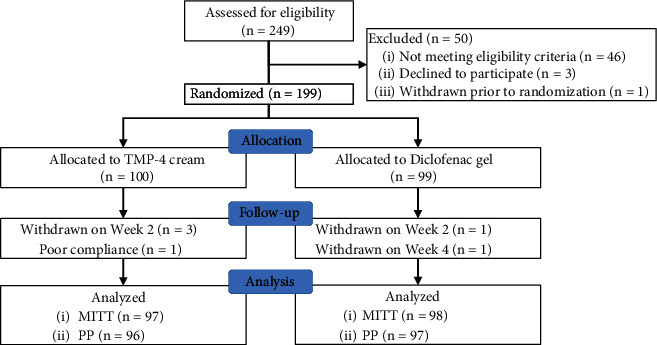
Flow diagram of the progress through all phases of this two-arm, randomized controlled trial (enrollment, intervention allocation, follow-up, and data analysis).

A total of 249 patients were initially assessed for eligibility, with 49 being excluded and one withdrawing her permission before the baseline evaluation at Week 0 owing to intolerable knee pain. One hundred and ninety-nine patients were randomly allocated to either the TMP-4 cream or diclofenac gel groups, with 100 receiving TMP-4 cream and 99 receiving diclofenac gel (Figure 2). The mean age of the participants was 61.4 ± 6.7 years (range: 46–83 years). The majority of the participants were females (*n* = 184, 92.5%) and had osteoarthritis in both knees (*n* = 159, 79.9%). The participants' baseline characteristics were comparable between the two groups, except for the duration of knee osteoarthritis which was longer in the TMP-4 group (5.1 ± 4.8 years) than in the diclofenac group (3.8 ± 2.8 years) (Table 1). During the first two weeks of the treatment phase, three participants in the TMP-4 group were withdrawn from the trial due to a flare-up of knee pain, a fever, and personal reasons, while one participant in the diclofenac group was withdrawn due to a drug allergy. During the last two weeks of the treatment phase, one participant who received diclofenac gel was withdrawn from the trial due to a flare-up of knee pain. One hundred and ninety-five participants (98.0%) were available for the MITT analysis, and 193 participants (97.0%) remained for the PP analysis (Figure 2).

### 3.1. Efficacy Assessment

**Figure 3:**
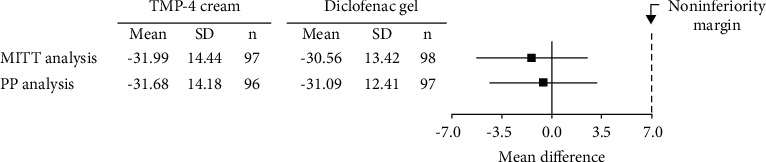
Noninferiority analysis of VAS knee pain. The upper limit of the two-sided 95% confidence interval (95% CI) for the mean difference of VAS knee pain did not surpass a margin of 7 mm in both MITT and PP analyses.

Concerning the primary endpoint, the mean change in VAS knee pain was not statistically significantly different between the two groups, and TMP-4 cream was shown to be noninferior to diclofenac gel in both MITT and PP analyses. The upper limit of the two-sided 95% CI for the comparison between the TMP-4 cream and diclofenac gel groups was within the prespecified margin of 7 mm for noninferiority (Figure 3).

At the end of the treatment phase, the participants in both groups showed a substantial improvement in all of the efficacy outcome measures (Figure S1 and Figure S2). The mean change in VAS knee stiffness, mSCT, TUG, and all the KOOS subscales from the baseline did not statistically significantly differ between the two groups (Table 2). There were 66 responders with TMP-4 cream compared to 62 with diclofenac gel (MITT analysis: RR = 1.075, 95% CI = 0.878–1.318, *p*=0.483; PP analysis: RR = 1.059, 95% CI = 0.864–1.298, *p*=0.579). Upon completion of the trial, the physician's assessment of overall improvement did not statistically significantly differ between the two groups (38.1 ± 14.9 vs. 41.3 ± 10.7, *p*=0.089); however, the patient's perception of overall improvement tended to favor diclofenac gel (65.1 ± 24.4 vs. 71.6 ± 19. 7, *p*=0.041).

### 3.2. Safety Assessment

During the four-week treatment phase, nine individuals experienced some adverse effects, all of which were nonserious. Six participants in the TMP-4 group reported nine adverse events: itching (*n* = 4), skin rash (*n* = 2), dyspepsia (*n* = 2), and skin redness (*n* = 1). Three participants in the diclofenac group experienced seven adverse events: itching (*n* = 2), skin rash (*n* = 1), burning sensation of skin (*n* = 1), dyspepsia (*n* = 1), palpitation (*n* = 1), and cystitis (*n* = 1). There were no statistically significant differences in any adverse outcomes between the two groups. In the TMP-4 group, there was no dropout owing to adverse drug reactions, but in the diclofenac group, there was one due to a drug allergy (*p*=0.497).

## 4. Discussion

In this four-week randomized controlled trial, topical treatment with either TMP-4 cream or diclofenac gel using a q.i.d. dosing schedule achieved analgesic efficacy with acceptable safety profiles in patients with symptomatic osteoarthritis of the knee. In both the MITT and PP analyses, the upper limit of 95% CI of MD in VAS knee pain was within the predetermined noninferiority margin of 7 mm, indicating that TMP-4 cream is noninferior to diclofenac gel in terms of knee pain relief. The absolute decrease in VAS knee pain after four weeks of treatment was approximately 31 mm on a horizontal 100 VAS in both groups; this finding can be considered a clinically significant improvement because it falls below the 19.9 mm cut-off value regarded as the minimum clinically meaningful improvement [56]. A changed value similar to this has been seen in previous studies with topical NSAIDs [45, 54]. The therapeutic effects of TMP-4 cream and diclofenac gel were further confirmed by categorical analysis of VAS knee pain, which revealed that about two-thirds of the participants in both groups experienced more than a 50% reduction in osteoarthritic knee pain after therapy. The proportion of those who responded to topical diclofenac was similar to a prior study [57]. Even though no other analgesic medicines were permitted during study participation, there was only one participant who prematurely dropped out from the trial, indicating that the therapies were effective. The overall data show that the majority of participants in both groups regarded their osteoarthritic knee pain to be clinically better. These findings support the efficacy of TMP-4 cream and diclofenac gel in osteoarthritic knee pain relief. Based on the results of this study, TMP-4 cream appears to be a viable alternative to topical diclofenac for the management of symptomatic osteoarthritis of the knee.

The present clinical trial examined symptoms of knee osteoarthritis using many distinct measures, allowing for a thorough assessment of the study intervention's efficacy [58, 59]. This study followed the OMERACT-OARSI core domain set of efficacy outcome measures, which included both patient-reported and objective outcome measures that are both valid, reliable, and responsive to change [60–62]. In all of the assessed efficacy outcome variables, topical application of TMP-4 was as effective as diclofenac gel, and mean changes from the baseline across all the efficacy outcome parameters did not significantly differ between the two groups over the four-week study period. All KOOS subscales were considerably higher in both groups, indicating that the physical function of the afflicted knee had improved [63]. Furthermore, the mSCT and TUG test results were much lower in both groups after four weeks of therapy, supporting that physical performance had improved [64]. These positive results are likely attributable to pain and stiffness reduction as a result of the intervention.

The mechanism of action of TMP-4 cream is broader than that of diclofenac gel or other NSAIDs and analgesics in current use for symptomatic osteoarthritis of the knee. Although the exact mechanisms of action have not yet been elucidated, the herbal components in the formulation have been demonstrated to exhibit a wide range of pharmacological actions. Based on the previous literature, *G*. *mangostana* extracts and their bioactive constituents (e.g., xanthones) exhibit a wide range of pharmacological activities, including antiinflammatory and antioxidant properties [65, 66]. A recent mouse experiment found that *α*-Mangostin, a xanthone derivative molecule derived from *G*. *mangostana* L. peel extract, might reduce inflammatory and oxidative responses, therefore alleviating the early clinical and histological signs of arthritis [67]. In numerous experimental models, *S*. *indicum* seed extracts have been found to exhibit antiinflammatory, antinociceptive, antioxidant, and chondroprotective effects [68–71]. Sesame oil is utilized in several topical pain therapies that have been clinically validated [30, 72–76]. Sesame seed supplementation given orally has been shown to reduce pain intensity and improve clinical signs and symptoms in patients with osteoarthritis of the knee [77]. *G*. *max* (L.) Merr. seed extracts and their bioactive constituents (e.g., genistein) have been shown to exhibit antinociceptive and antiinflammatory properties [78, 79]. *C*. *asiatica* leaf extracts and their bioactive components (e.g., madecassic acid) have been shown to produce analgesic, antiinflammatory, and cartilage-protective effects in both *in vitro* and *in vivo* models [80–82]. Based on the above-mentioned evidence, the positive results of TMP-4 therapy are of no surprise from a pathophysiologic point of view, given low-grade chronic inflammation is the primary cause of osteoarthritis development [83]. TMP-4's benefits in symptomatic alleviation of osteoarthritic knee pain may be due to the combination and perhaps synergistic pharmacological actions of the various herbal components in the formulation.

TMP-4 cream was well tolerated after four weeks of therapy, with a safety profile similar to diclofenac gel. There were no concerns about the drug's safety as the majority of the adverse events reported in this study were minor and localized. Skin reactions on the application site accounted for two-thirds of the reported events, which is consistent with the literature [84]. These findings are not surprising given the low systemic absorption of topical formulations compared to oral formulations [85].

The results of the present study should be viewed in light of the study's limitations. First and foremost, there was no placebo-controlled group in this study. In a situation where topical NSAIDs are commonly prescribed and available, delaying effective therapy may not be deemed ethical. Second, the test intervention (TMP-4 cream) did not appear to be identical to the comparator (diclofenac gel). Although neither the outcome assessors nor the participants were told of the treatment assignment, color and/or texture variations between the two interventions might have alerted the participants to the intervention they and the other participants were allocated in this study. This might put the trial's blind assignment in jeopardy and add performance and/or detection bias to herbal medicine research whereby it is challenging to find a comparator that is exactly like the test intervention. Third, even though the VAS is accurate and reliable for pain assessment, it is subjective and heavily reliant on the patient's perception of pain [86]. It is well documented that certain levels of pain alleviation following a therapeutic intervention might be largely attributable to contextual features, including patient attitudes and expectations, as well as the patient-physician interaction [87, 88]. However, this study included various efficacy outcome measures, some of which had fewer contextual effects than pain measurements, thereby reducing the risk of subjective result assessment bias [89].

Last but not the least limitation, the promising results of TMP-4 cream should not be extended beyond the four-week time frame being studied in this trial. The intervention's short-term effect is consistent with numerous prior studies signifying topical diclofenac's efficacy in the first few weeks [90–92]. Although several clinical trials have demonstrated that topical NSAIDs may be effective in osteoarthritic knee pain management over 12 weeks of treatment [93–97], there are currently insufficient data to support the use of topical medicines for long-term pain management in patients with osteoarthritis of the knee [98, 99]. More research is needed to determine the long-term effectiveness and safety of TMP-4 cream in the management of symptomatic osteoarthritis of the knee, as this chronic and degenerative condition necessitates long-term therapy.

## 5. Conclusions

In this randomized controlled trial, TMP-4 cream applied four times daily was found to be noninferior to diclofenac gel in alleviating osteoarthritic knee pain. In addition, TMP-4 cream was as effective as diclofenac gel in terms of improving key efficacy outcomes of knee osteoarthritis. TMP-4 cream may be considered a viable alternative to topical diclofenac in the management of symptomatic osteoarthritis of the knee.

## Figures and Tables

**Table 1:** Participants' demographic and clinical features.

	TMP-4 cream group (*n* = 100)	Diclofenac gel group (*n* = 99)
Age (years)	62.1 ± 6.8	60.7 ± 6.4
Gender (female: male)	95 : 5	89 : 10
Body mass index (kg/m^2^)	27.0 ± 4.2	26.5 ± 4.2
Localization of knee osteoarthritis (right knee : left knee : both knees)	12 : 9 : 79	12 : 7 : 80
Kellgren–Lawrence grade (right knee : left knee)		
Grade 2	36 : 34	35 : 31
Grade 3	39 : 39	30 : 35
Grade 4	16 : 15	27 : 21
Duration of knee osteoarthritis (year)	5.1 ± 4.8	3.8 ± 2.8
Baseline efficacy outcome measures		
VAS knee pain	54.0 ± 11.5	52.5 ± 8.3
VAS knee stiffness	47.8 ± 18.6	46.0 ± 18.1
KOOS		
Pain	52.7 ± 13.7	53.5 ± 14.7
Other knee symptoms	58.2 ± 15.0	59.7 ± 14.5
Activities of daily living	53.0 ± 14.7	52.8 ± 14.9
Sport and recreation function	26.2 ± 17.3	27.0 ± 17.3
Knee-related quality of life	32.6 ± 15.2	32.3 ± 14.6
mSCT (sec)	12.7 ± 8.4	11.3 ± 5.4
TUG (sec)	15.8 ± 8.0	15.3 ± 4.8

KOOS: Knee Injury and Osteoarthritis Outcome Score; mSCT: modified 10-step stair climb test; TMP-4: Thai Medicinal Plant-4; TUG, timed up and go test; VAS: Visual Analog Scale.

**Table 2:** Efficacy outcome assessments at the end of the treatment phase.

	TMP-4 cream	Diclofenac gel	MD	(95% CI)	*p* value^a^
Mean changes in VAS knee pain					
MITT analysis	−31.99 ± 14.44	−30.56 ± 13.42	−1.43	(−5.37 to 2.51)	0.475
PP analysis	−31.68 ± 14.18	−31.09 ± 12.41	−0.58	(−4.37 to 3.20)	0.761
Mean changes in VAS knee stiffness					
MITT analysis	−26.27 ± 16.75	−25.46 ± 15.58	−0.81	(−5.38 to 3.76)	0.727
PP analysis	−26.17 ± 16.81	−25.90 ± 15.05	−0.27	(-4.80 to 4.26)	0.907
Mean changes in mSCT					
MITT analysis	−3.95 ± 6.76	−3.26 ± 4.13	−0.69	(−2.27 to 0.89)	0.388
PP analysis	−3.95 ± 6.80	−3.30 ± 4.13	−0.65	(−2.24 to 0.95)	0.423
Mean changes in TUG					
MITT analysis	−3.61 ± 5.99	−3.29 ± 3.56	−0.32	(−1.71 to 1.07)	0.648
PP analysis	−3.63 ± 6.02	−3.33 ± 3.55	−0.30	(−1.70 to 1.11)	0.678
Mean changes in KOOS pain					
MITT analysis	18.19 ± 18.39	19.11 ± 18.16	−0.93	(−6.09 to 4.24)	0.724
PP analysis	18.11 ± 18.47	19.39 ± 18.04	−1.28	(−6.46 to 3.91)	0.628
Mean changes in KOOS other symptoms					
MITT analysis	17.55 ± 16.41	17.03 ± 16.10	0.52	(−4.08 to 5.11)	0.516
PP analysis	17.44 ± 16.46	17.13 ± 16.15	0.30	(−4.33 to 4.93)	0.897
Mean changes in KOOS activities of daily living					
MITT analysis	18.42 ± 17.13	21.34 ± 15.91	−2.91	(−7.58 to 1.76)	0.220
PP analysis	18.16 ± 17.02	21.54 ± 15.87	−3.38	(−8.05 to 1.29)	0.155
Mean changes in KOOS sport and recreation function					
MITT analysis	19.69 ± 21.80	22.50 ± 20.97	−2.81	(−8.85 to 3.23)	0.360
PP analysis	19.48 ± 21.82	22.84 ± 20.82	−3.36	(−9.41 to 2.70)	0.276
Mean changes in KOOS knee-related quality of life					
MITT analysis	15.45 ± 20.16	19.48 ± 16.84	−4.03	(−9.27 to 1.22)	0.132
PP analysis	15.29 ± 20.20	20.07 ± 15.86	−4.78	(−9.94 to 0.38)	0.069

^a^Student's *t*-test. CI: confidence interval; KOOS: Knee Injury and Osteoarthritis Outcome Score; MD: mean difference; MITT: modified intention-to-treat; mSCT: modified 10-step stair climb test; PP: per-protocol; TMP-4: Thai Medicinal Plant-4; TUG: timed up and go test; VAS: Visual Analog Scale.

## Data Availability

The datasets used and/or analysed during the current study are available from the corresponding author on reasonable request.

## Supplementary Materials

Supplementary Materials Table S1: The components of Thai Medicinal Plants-4 (TMP-4) cream. Figure S1: VAS pain, VAS stiffness, mSCT, and TUG at baseline, Week 2, and Week 4. Results are presented as the mean ± standard deviation: (a) MITT analysis; (b) PP analysis. ^*∗*^Statistically significantly difference between the two groups. Figure S2: KOOS at the baseline, Week 2, and Week 4. Results are presented as the mean ± standard deviation: (a) MITT analysis; (b) PP analysis. ^*∗*^Statistically significantly difference between the two groups.
