# Supplementary material for: A Randomized Controlled Trial of Thai Medicinal Plant-4 Cream versus Diclofenac Gel in the Management of Symptomatic Osteoarthritis of the Knee
Source: Evid Based Complement Alternat Med. 2022 Jun 13;2022:8657000. doi: 10.1155/2022/8657000 (PMC9208949; doi:10.1155/2022/8657000)
Supplement: Supplementary Materials — Table S1: The components of Thai Medicinal Plants-4 (TMP-4) cream. Figure S1: VAS pain, VAS stiffness, mSCT, and TUG at baseline, Week 2, and Week 4. Results are presented as the mean ± standard deviation: (a) MITT analysis; (b) PP analysis. ∗Statistically significantly difference between the two groups. Figure S2: KOOS at the baseline, Week 2, and Week 4. Results are presented as the mean ± standard deviation: (a) MITT analysis; (b) PP analysis. ∗Statistically significantly difference between the two groups. [file 8657000.f1.zip › 8657000.f1/Figure S2 (1).pdf]

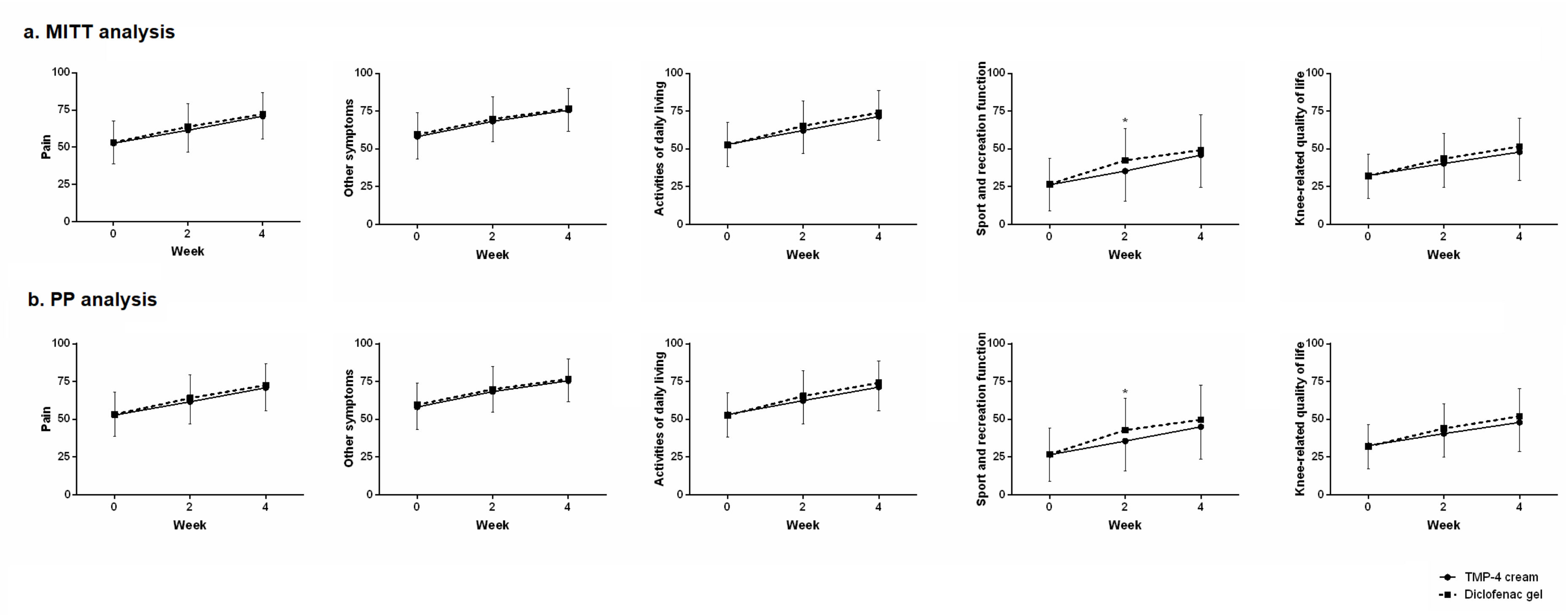

Figure S2: KOOS at baseline, Week 2, and Week 4. Results are presented as mean ± standard deviation: (a) MITT analysis; (b) PP analysis. *Statistically significantly different between the two groups.
